# LDL-cholesterol to HDL-cholesterol ratio discordance with lipid parameters and carotid intima-media thickness: a cohort study in China

**DOI:** 10.1186/s12944-020-01324-5

**Published:** 2020-06-18

**Authors:** Yanmei Lou, Xue Li, Liming Cao, Pei Qin, Jing Shi, Yanyan Zhang, Changyi Wang, Jianping Ma, Li Wang, Xiaolin Peng, Hongen Chen, Shan Xu, Fulan Hu, Yashuang Zhao, Ping Zhao

**Affiliations:** 1 Department of Health Management, Beijing Xiao Tang Shan Hospital, Beijing, People’s Republic of China; 2 Department of Epidemiology, College of Public Health, Harbin Medical University, Harbin, Heilongjiang People’s Republic of China; 3 School of Public Health, Shenzhen University Health Science Center, Shenzhen, Guangdong People’s Republic of China; 4 Department of Non-communicable Disease Prevention and Control, Shenzhen Nanshan Center for Chronic disease, Shenzhen, Guangdong People’s Republic of China

**Keywords:** Carotid intima-media thickness, Low-density lipoprotein cholesterol/high-density lipoprotein cholesterol radio, Discordance, Low-density lipoprotein cholesterol, High-density lipoprotein cholesterol, Total cholesterol, Triglycerides, Cohort

## Abstract

**Background:**

The discordance of the low-density lipoprotein cholesterol-to-high-density lipoprotein cholesterol (LDL-C/HDL-C) ratio with alterative lipid parameters may explain the inconsistent association of CIMT with the LDL-C/HDL-C ratio. Therefore, this study aimed to explore the associations between LDL-C/HDL-C ratio discordance with alternative lipid parameters and elevated carotid intima-media thickness (CIMT) risk in a large cohort in Beijing, China.

**Methods:**

In total, 13,612 adults who didn’t have elevated CIMT at baseline and who participated in at least one follow-up of annual examination between 2009 and 2016 were included in this cohort study. A multivariable Cox regression model was utilized to evaluate the associations of discordance of the LDL-C/HDL-C ratio with TC, TGs, LDL-C and HDL-C with elevated CIMT risk.

**Results:**

During 37,999 person-years of follow-up, 2004 individuals (1274 men and 730 women) developed elevated CIMT. Among individuals with normal TC and TGs, 16.6 and 15.2% individuals had a discordantly high LDL-C/HDL-C ratio, respectively, and the risk of elevated CIMT increased by 1.54 (95% CI 1.33, 1.77) and 1.53 (95% CI 1.33, 1.76), respectively, comparing to individuals with a concordantly low LDL-C/HDL-C ratio. A high LDL-C/HDL-C ratio could significantly increase elevated CIMT risk regardless of discordance/concordance with LDL-C and HDL-C (*P* < 0.001). A low LDL-C/HDL-C ratio with discordantly normal HDL-C and high LDL-C (13.2% of individuals) had a 32% (HR = 1.32, 95% CI 1.11, 1.57) higher risk of elevated CIMT than concordantly low LDL-C and normal HDL-C. Sensitivity analysis by excluding CIMT developed in the first 2 years follow-up further confirmed the above results.

**Conclusions:**

A high LDL-C/HDL-C ratio could significantly increase elevated CIMT risk regardless of discordance/concordance with TC, TGs, LDL-C and HDL-C Even a low LDL-C/HDL-C ratio with discordantly high LDL-C and normal HDL-C could also significantly increase CIMT risk. Individuals should maintain both the LDL-C/HDL-C ratio and LDL-C at normal levels to prevent elevated CIMT.

## Introduction

Carotid intima-media thickness (CIMT), an alternative indicator of subclinical atherosclerosis, can predict future ischemic cardiovascular disease (CVD) and stroke events [1–5]. Approximately 30% of first acute cardiovascular events without any clinical symptoms are fatal [6]. Thus, identifying risk factors for the silent preclinical stage of CIMT is helpful to prevent CVDs and stroke.

High-density lipoprotein cholesterol (HDL-C) is anti-atherogenic [7]. Low-density lipoprotein cholesterol (LDL-C) is atherogenic and a dominating target of lipid-lowering treatment [7]. However, despite LDL-C lowering treatment, CVD events continue to occur [8, 9]. The LDL-C to HDL-C (LDL-C/HDL-C) ratio may provide additional information and has been suggested to be superior to single HDL-C and LDL-C in predicting atherosclerosis progression and CVD [10, 11]. Previous studies have reported significant association of CIMT with the LDL-C/HDL-C ratio [12, 13]. However, other studies failed to identify a significant relation of CIMT with the LDL-C/HDL-C ratio [14, 15]. Discordance of the LDL-C/HDL-C ratio with alternative lipid parameters may exist in the clinical setting, and it might be helpful for clinicians to estimate the relationships of the ratio of LDL-C/HDL-C discordance with various lipid parameters with elevated CIMT.

Therefore, this study was conducted to explore the relations between the ratio of LDL-C/HDL-C discordance with alternative lipid parameters and elevated CIMT risk in a large cohort in Beijing, China.

## Materials and methods

### Participants

From 2009 to 2016, individuals who were diagnosed without increased CIMT or carotid plaque at the baseline health examination and who participated at least one follow-up health check-up at Xiaotangshan Hospital were included and formed the cohort assessed in the current study (*n* = 16,464). Individuals with cancer (*n* = 104), coronary heart disease (*n* = 112), stroke (*n* = 33), myocardial infarction (n = 11), or missing CIMT data (*n* = 37) at baseline or carotid plaque data (*n* = 2470) at follow-up were excluded from the current study. In addition, 32 individuals aged < 20 or ≥ 80 years old at baseline were further excluded. Finally, 13,681 participants entered into this study. The Institutional Review Board of the Xiaotangshan Hospital approved this study (No. 202006). Only conventional health examination information was used for data analysis, so the requirement of informed consent from participants was waived.

### Carotid artery color Doppler ultrasound

A 3.5-*MHz* transducer (logic Q700 MR, GE, Milwaukee, WI, USA) was used to perform vascular ultrasonography on the right and left carotid arteries by experienced radiologists. Ultrasonographic images of the common carotid artery was recorded by a frequency of 5–10 *MHz*; the carotid artery bulb and proximal internal carotid arteries ultrasonographic images were acquired by a frequency 9 *MHz*. For the sake of reducing variability of cardiac cycle, the diastolic images were recorded for all the ultrasonographic images.

The distance from the echo front of the lumen-intima to the echo front of the media adventitia was quantified as carotid IMT for the bifurcation of carotid artery and arteria carotis communis [16]. IMT value was defined as the maximum IMT of the carotid bifurcation and bilateral arteria carotis communis. Elevated IMT was defined as IMT ≥1 mm [17].

### LDL-C/HDL-C ratio criteria

Peripheral venous blood fasting for more than 12 h were obtained from all subjects in the morning. Serum levels of triglycerides (TGs), total cholesterol (TC), HDL-C, LDL-C and creatinine were detected by enzymatic colorimetry on the Roche Cobas C 710 automatic biochemical analyzer (Supplied by Beijing Barry Medical Equipment Co., Ltd., Beijing, China) according to the instructions of the manufacturer. TC, TGs, HDL-C and LDL-C kits, and lip.c Calibrators were provided by Roche (Beijing Barry Medical Equipment Co., Ltd., Beijing, China), and the quality control products PUND and PNUX were provided by Bio-Rad (supplied by Beijing Jingyang Tenghui Technology Development Co., Ltd., Beijing, China).

Participants were categorized into four groups by quartiles (Q1–4) of LDL-C/HDL-C ratio at baseline: ≤1.63, 1.63 to 2.13, 2.13 to 2.70, and > 2.70. The optimal threshold of the LDL-C/HDL-C ratio was 2.47, determined by the area under the receiver operating characteristic (ROC) curve (AUC).

Discordance was defined as an LDL-C/HDL-C ratio > 2.47 and TGs < 1.70 mmol/L, LDL-C/HDL-C ratio > 2.47 and TC < 5.18 mmol/L, LDL-C/HDL-C ratio > 2.47 and HDL-C ≥ 1.04 mmol/L, LDL-C/HDL-C ratio > 2.47 and LDL-C < 3.37 mmol/L, or vice versa.

### Covariate assessment

At baseline and at every follow-up, information on age; sex; medical history of CVD, stroke and cancer; and use of medications was collected from all participants. Anthropometric measurements included waist circumference (WC), weight, and height. BMI was calculated as [weight (kg)]/[height (meters)^2^]. The electronic sphygmomanometer (HEM-770AFuzzy, Omron, Japan) was used to meter systolic and diastolic blood pressure. The glucose dehydrogenase method was used to measure fasting plasma glucose (FPG) (Merck, Darmstadt, Germany).

Hypertension was diagnosed according to a systolic blood pressure ≥ 140 mmHg, diastolic blood pressure ≥ 90 mmHg, or taking anti-hypertension medicine [18]. T2DM was diagnosed according to a fasting plasma glucose (FPG) level ≥ 126 mg/dL, 2-h glucose ≥200 mg/dL, HbA1c ≥6.5% or taking anti-diabetes medicine [19]. Fatty liver disease diagnosis was performed according to the guidelines described by the Chinese Liver Disease Association [20].

### Statistical analyses

Participants’ baseline characteristics are described based on quartiles of the LDL-C/HDL-C ratio at baseline. Frequency (%) was used to describe qualitative variables, and the mean ± SD (standard deviation) or median (25% ~ 75%) was used to present quantitative variables.

The study endpoint was elevated CIMT formation. Follow-up person-years were calculated from the first entry date to the last confirmed follow-up date or the elevated CIMT formation date. Three Cox regression models were established to estimate the associations of discordance of the LDL-C/HDL-C ratio with TC, TGs, LDL-C, and HDL-C with elevated CIMT risk. Hazard ratios (HRs) and 95% confidence intervals (CIs) were calculated in 3 models: Model 1 was adjusted for sex and baseline age; Model 2 was further adjusted for WC, TC, TGs, creatinine, BMI, smoking, alcohol consumption, lipid-lowering medications, T2DM, hypertension, and fatty liver disease at baseline; and Model 3 was further adjusted for white blood cell count (WBC) at baseline and all covariates in Model 2.

To assess the robustness of the results, sensitivity analyses were conducted by removing incident elevated CIMT individuals occurring in the first two years of follow-up. Subgroup analyses were also conducted by stratification according to age, sex, hypertension and T2DM. For discordance of the LDL-C/HDL-C ratio with LDL-C level and HDL-C level, subgroup analysis stratified by T2DM was not conducted because of the small sample size of T2DM at baseline but sensitivity analysis was conducted by removing T2DM participants at baseline. Statistically significant was considered with a 2-sided *P* < 0.05, all statistical analyses were performed with R 3.5.2 (R Foundation, Boston, MA).

## Results

During 37,999 person-years of follow-up, 2004 individuals (1274 men; 730 women) developed elevated CIMT; the incidence rates (per 1000 person-years) were 52.77 overall, 60.2 in men and 42.30 in women. The median age was 41 years (range 20–78), and 47.4% of the participants were women. The individuals’ baseline characteristics are shown in Table 1.

**Table 1:** Baseline characteristics of the study participants according to baseline LDL-C/HDL-C ratio quartiles

Baseline characteristics	LDL-C/HDL-C ratio quartiles^a^				*P value*
	~ 1.68 (*n* = 3405)	1.69 ~ 2.20 (n = 3405)	2.21 ~ 2.78 (*n* = 3408)	2.79 ~ (*n* = 3394)	
**Total**					
Age (years)					< 0.001
< 45	2341	2020	1906	1956	
≥ 45	1064	1385	1502	1438	
Sex					< 0.001
Men	988	1591	2120	2502	
Women	2417	1814	1288	892	
Height (cm)	164.33 ± 7.54	166.06 ± 8.04	167.92 ± 8	169.3 ± 7.65	< 0.001
Weight (kg)	61.73 ± 11.18	67.7 ± 11.95	72.69 ± 12.18	76.73 ± 12.23	< 0.001
BMI (kg/m^2^)	22.76 ± 3.17	24.45 ± 3.27	25.67 ± 3.25	26.68 ± 3.23	< 0.001
Waist (cm)	75.88 ± 9.48	81.57 ± 9.84	85.68 ± 9.53	89.07 ± 9.03	< 0.001
Heart rate (beats/min)	76.65 ± 10.45	76.75 ± 10.31	76.98 ± 10.14	78.45 ± 10.8	< 0.001
SUA (mmol/L)	278.92 ± 75.57	310.79 ± 80.95	341.45 ± 84.64	364.35 ± 84.22	< 0.001
Creatinine (μmol/L)	70.49 ± 19.59	75.22 ± 22.18	78.34 ± 15.76	79.61 ± 15.59	< 0.001
TC (mmol/L)	4.24 ± 0.77	4.58 ± 0.75	4.85 ± 0.78	5.32 ± 0.85	< 0.001
TG (mmol/L) (median [range])	0.75(0.23–17.70)	1.02(0.30–18.41)	1.37(0.39–17.85)	1.73(0.43–15.81)	< 0.001
ALT (U/L)	17.62 ± 12.27	21.31 ± 18.76	24.71 ± 17.11	30.06 ± 22.8	< 0.001
AST (U/L)	19.29 ± 8.09	20.56 ± 10.76	21.27 ± 8.79	22.95 ± 10.41	< 0.001
Lipid lowering treatment					0.039
No	3395	3394	3384	3380	
Yes	10	11	24	14	
Smoking					< 0.001
Never	2731	2415	2184	1991	
Ever	313	485	566	650	
Current	361	504	658	753	
Drinking					< 0.001
Never	2574	2204	1808	1597	
Ever	265	336	400	479	
Current	566	865	1200	1318	
Hypertension					< 0.001
No	3081	2854	2671	2522	
Yes	324	551	737	872	
T2DM					< 0.001
No	3331	3273	3216	3143	
Yes	74	132	192	251	
Fatty liver					< 0.001
No	3025	2532	1972	1400	
Yes	379	869	1435	1992	

*Abbreviation*: *BMI* body mass index, *WC* waist circumference, *SUA* serum uric acid, *TC* total cholesterol, *TG* triglycerides, *ALT* alanine aminotransferase, *AST* glutamic oxaloacetic transaminase

^a^missing data for LDL-C_HDL-C ratio: 69

### Discordance of the LDL-C/HDL-C ratio with TC, and TGs and elevated CIMT risk

Among individuals with normal TC, 16.6% of individuals had a discordantly high LDL-C/HDL-C ratio, with a 54% (HR = 1.54, 95% CI 1.33–1.77) higher risk of elevated CIMT than that among individuals who had a concordantly low ratio of LDL-C/HDL-C according to model 3. In contrast, there is no significant association between a low ratio of LDL-C/HDL-C with discordantly high TC (20.2% individuals) and elevated CIMT risk. The sensitivity analyses by removing incident elevated CIMT during the first two years of follow-up further confirmed the above results (Table 2). In the subgroup analyses, the association between the discordance of a high ratio of LDL-C/HDL-C with low TC and elevated CIMT risk remained significant in all the subgroups except the subgroups of women (HR = 1.02, 95% CI 0.69, 1.49) and participants with a BMI ≤ 24 (HR = 1.35, 95% CI 0.97, 1.88) in model 3, with the highest HR of 1.77 (95% CI 1.09, 2.89) in T2DM patients. Details are shown in Suppl. Table 1.

**Table 2:** Association between LDL-C/HDL-C ratio concordance with TC and CIMT risk

	No. of participants/cases	Model 1^a^ (HR (95%CI))	Model 2^b^ (HR (95%%CI))	Model 3^c^ (HR (95%%CI))
**Total**				
Concordantly low LDL-C/HDL-C ratio and low TC	8119/923	1	1	1
Discordantly low LDL-C/HDL-C ratio and high TC	2060/383	1.00 (0.89, 1.13)	1.04 (0.92, 1.17)	1.04 (0.92, 1.18)
Discordantly high LDL-C/HDL-C ratio and low TC	1620/269	1.59 (1.38, 1.83)	1.55 (1.34, 1.78)	1.54 (1.33, 1.77)
Concordantly high LDL-C/HDL-C ratio and high TC	1812/420	1.66 (1.47, 1.86)	1.65 (1.45, 1.87)	1.64 (1.45, 1.86)
**Sensitivity analysis by excluding CIMT in the first 2 years**				
Concordantly low LDL-C/HDL-C ratio and low TC	7915/719	1	1	1
Discordantly low LDL-C/HDL-C ratio and high TC	2013/336	1.06 (0.93, 1.21)	1.09 (0.95, 1.24)	1.1 (0.96, 1.25)
Discordantly high LDL-C/HDL-C ratio and low TC	1561/210	1.65 (1.41, 1.93)	1.60 (1.37, 1.89)	1.60 (1.36, 1.87)
Concordantly high LDL-C/HDL-C ratio and high TC	1720/328	1.63 (1.42, 1.86)	1.61 (1.4, 1.85)	1.61 (1.40, 1.85)

^a^Model 1: adjusted for age, gender at baseline

^b^Model 2: adjusted for age, gender, BMI, smoking, drinking, WC, TC, TG, creatinine, lipid-lowering medications, T2DM, hypertension, and fatty liver at baseline

^c^Model 3: adjusted for age, gender, BMI, smoking, drinking, WC, TC, TG, creatinine, WBC, lipid-lowering medications, T2DM, hypertension, and fatty liver at baseline

Among individuals with normal TGs, the probability of elevated CIMT was increased (HR = 1.53, 95% CI 1.33, 1.76) in participants with a discordantly high ratio of LDL-C/HDL-C (15.2%) comparing to participants with a concordantly low ratio of LDL-C/HDL-C in model 3. In contrast, there is no significant association between a low ratio of LDL-C/HDL-C with discordantly high TGs (17.6% individuals) and elevated CIMT risk. The sensitivity analyses by removing incident elevated CIMT individuals during the first two years of follow-up showed consistent results (Table 3). In the subgroup analyses, discordance of a high ratio of LDL-C/HDL-C with low TGs and elevated CIMT risk remained significant for all the subgroups except that of T2DM (HR = 1.34, 95% CI 0.77, 2.32) in model 3. The HR decreased in the hypertension (1.41, 95% CI 1.07, 1.86) and BMI > 24 (1.39, 95% CI 1.20, 1.61) subgroups (Suppl. Table 2).

**Table 3:** Association between LDL-C/HDL-C ratio concordance with TG and CIMT risk

	No. of participants/cases	Model 1^a^ (HR (95%CI))	Model 2^b^ (HR (95%%CI))	Model 3^c^ (HR (95%%CI))
**Total**				
Concordantly low LDL-C/HDL-C ratio and low TG	8208/995	1	1	1
Discordantly low LDL-C/HDL-C ratio and high TG	1971/311	0.98 (0.86, 1.11)	0.86 (0.75, 0.99)	0.85 (0.74, 0.97)
Discordantly high LDL-C/HDL-C ratio and low TG	1648/309	1.68 (1.48, 1.92)	1.56 (1.36, 1.79)	1.53 (1.33, 1.76)
Concordantly high LDL-C/HDL-C ratio and TG	1784/380	1.57 (1.39, 1.78)	1.37 (1.19, 1.57)	1.34 (1.17, 1.55)
**Sensitivity analysis by excluding CIMT in the first 2 years**				
Concordantly low LDL-C/HDL-C ratio and low TG	8004/719	1	1	1
Discordantly low LDL-C/HDL-C ratio and high TG	1924/336	1.01 (0.88, 1.16)	0.89 (0.77, 1.03)	0.88 (0.76, 1.02)
Discordantly high LDL-C/HDL-C ratio and low TG	1577/210	1.69 (1.46, 1.96)	1.6 (1.37, 1.86)	1.57 (1.35, 1.83)
Concordantly high LDL-C/HDL-C ratio and TG	1704/328	1.55 (1.35, 1.78)	1.36 (1.17, 1.58)	1.36 (1.17, 1.58)

^a^Model 1: adjusted for age, gender at baseline

^b^Model 2: adjusted for age, gender, BMI, smoking, drinking, WC, TC, TG, creatinine, lipid-lowering medications, T2DM, hypertension, and fatty liver at baseline

^c^Model 3: adjusted for age, gender, BMI, smoking, drinking, WC, TC, TG, creatinine, WBC, lipid-lowering medications, T2DM, hypertension, and fatty liver at baseline

### Discordance of the LDL-C/HDL-C ratio with LDL-C level and HDL-C level and elevated CIMT risk

As shown in Table 4, among individuals with a low ratio of LDL-C/HDL-C, the probability of elevated CIMT was significantly associated with a low ratio of LDL-C/HDL-C with discordantly high LDL-C level and normal HDL-C level (13.2% individuals) (HR = 1.32 95% CI 1.11, 1.57) comparing to concordantly low LDL-C level and normal HDL-C level, despite no association of elevated CIMT probability with a low ratio of LDL-C/HDL-C with discordantly low LDL-C level and abnormal HDL-C level (6.7% of individuals).

**Table 4:** Association between LDL-C/HDL-C ratio concordance with LDL-C, HDL-C and CIMT risk

	No. of participants/cases	Model 1^a^ (HR (95%CI))	Model 2^b^ (HR (95%%CI))	Model 3^c^ (HR (95%%CI))
**Total**				
Concordantly low LDL-C/HDL-C ratio, low LDL-C and normal HDL-C	8158/977	1	1	1
Discordantly low LDL-C/HDL-C ratio, high LDL-C and normal HDL-C	1343/246	1.21 (1.05, 1.39)	1.33 (1.12, 1.59)	1.32 (1.11, 1.57)
Discordantly low LDL/HDL-C ratio, low LDL-C and abnormal HDL-C	679/84	1.08 (0.86, 1.35)	1.01 (0.8, 1.27)	0.99 (0.79, 1.25)
Discordantly high LDL/HDL-C ratio, low LDL-C and normal HDL-C	472/77	1.46 (1.15, 1.84)	1.43 (1.13, 1.81)	1.43 (1.13, 1.81)
Discordantly high LDL/HDL-C ratio, low LDL-C and abnormal HDL-C	749/137	1.54 (1.29, 1.85)	1.49 (1.23, 1.8)	1.48 (1.22, 1.79)
Discordantly high LDL/HDL-C ratio, high LDL-C and normal HDL-C	1793/401	1.74 (1.55, 1.96)	1.84 (1.58, 2.13)	1.82 (1.56, 2.11)
Concordantly high LDL/HDL-C ratio, high LDL-C and abnormal HDL-C	418/74	2.08 (1.63, 2.64)	1.97 (1.52, 2.54)	1.87 (1.44, 2.42)
**Sensitivity analysis by excluding CIMT in the first 2 years**				
Concordantly low LDL-C/HDL-C ratio, low LDL-C and normal HDL-C	8158/789	1	1	1
Discordantly low LDL-C/HDL-C ratio, high LDL-C and normal HDL-C	1343/204	1.21 (1.03, 1.41)	1.21 (1.04, 1.42)	1.22 (1.04, 1.43)
Discordantly low LDL/HDL-C ratio, low LDL-C and abnormal HDL-C	679/62	1.01 (0.78, 1.31)	0.95 (0.73, 1.24)	0.92 (0.70, 1.21)
Discordantly high LDL/HDL-C ratio, low LDL-C and normal HDL-C	472/61	1.45 (1.12, 1.89)	1.44 (1.1, 1.87)	1.42 (1.09, 1.85)
Discordantly high LDL/HDL-C ratio, low LDL-C and abnormal HDL-C	749/105	1.49 (1.21, 1.83)	1.41 (1.14, 1.75)	1.39 (1.12, 1.73)
Discordantly high LDL/HDL-C ratio, high LDL-C and normal HDL-C	1793/319	1.72 (1.51, 1.97)	1.69 (1.47, 1.94)	1.69 (1.47, 1.94)
Concordantly high LDL/HDL-C ratio, high LDL-C and abnormal HDL-C	418/53	2.01 (1.51, 2.66)	1.83 (1.36, 2.46)	1.75 (1.30, 2.35)
**Sensitivity analysis by excluding T2DM at baseline**				
Concordantly low LDL-C/HDL-C ratio, low LDL-C and normal HDL-C	7882/910	1	1	1
Discordantly low LDL-C/HDL-C ratio, high LDL-C and normal HDL-C	1287/238	1.23 (1.07, 1.42)	1.22 (1.05, 1.41)	1.22 (1.06, 1.42)
Discordantly low LDL/HDL-C ratio, low LDL-C and abnormal HDL-C	616/72	1.06 (0.83, 1.35)	1.04 (0.81, 1.33)	1.02 (0.79, 1.31)
Discordantly high LDL/HDL-C ratio, low LDL-C and normal HDL-C	441/71	1.49 (1.17, 1.9)	1.50 (1.17, 1.91)	1.50 (1.17, 1.91)
Discordantly high LDL/HDL-C ratio, low LDL-C and abnormal HDL-C	696/120	1.50 (1.23, 1.82)	1.45 (1.19, 1.78)	1.45 (1.18, 1.77)
Discordantly high LDL/HDL-C ratio, high LDL-C and normal HDL-C	1662/369	1.77 (1.56, 2.00)	1.74 (1.53, 1.97)	1.73 (1.52, 1.96)
Concordantly high LDL/HDL-C ratio, high LDL-C and abnormal HDL-C	379/61	1.93 (1.49, 2.51)	1.85 (1.41, 2.43)	1.80 (1.37, 2.37)

^a^Model 1: adjusted for age, gender at baseline

^b^Model 2: adjusted for age, gender, BMI, smoking, drinking, WC, TC, TG, creatinine, lipid-lowering medications, T2DM, hypertension, and fatty liver at baseline

^c^Model 3: adjusted for age, gender, BMI, smoking, drinking, WC, TC, TG, creatinine, WBC, lipid-lowering medications, T2DM, hypertension, and fatty liver at baseline

Among individuals with a high ratio of LDL-C/HDL-C, discordantly normal LDL-C level and low HDL-C level existed in13.2% of individuals, discordantly abnormal HDL-C level and low LDL-C level existed in 21.8% of individuals, discordantly normal HDL-C level and high level of LDL-C existed in 52.2% of individuals, and concordantly abnormal HDL-C level and high level of LDL-C existed in 12.2% of individuals. Compared with those with concordantly normal HDL-C level and low LDL-C level, the corresponding HR in model 3 was highest in those with concordantly abnormal HDL-C level and high level of LDL-C (1.87 95% CI 1.44, 2.42), followed by those with discordantly and normal HDL-C level and high level of LDL-C (1.82 (95% CI 1.56, 2.11)), those with discordantly abnormal HDL-C level and low LDL-C level (1.48 (95% CI 1.22, 1.79)), and those with discordantly normal LDL-C level and low HDL-C level (1.43 (95% CI 1.13, 1.81)).

The sensitivity analyses by removing incident elevated CIMT individuals that occurred in the first two years of follow-up and removing individuals with T2DM at baseline further confirmed the above results (Table 4). In the subgroup analyses, the association between a discordantly low ratio of LDL-C/HDL-C with high LDL-C level and normal HDL-C level and elevated CIMT risk became nonsignificant in the subgroups of women, participants aged > 45 years, participants with a BMI ≤ 24, and participants with hypertension in Model 3. A discordantly high ratio of LDL/HDL-C with normal HDL-C and low LDL-C levels was not significantly related with elevated CIMT risk in the subgroups of women, participants aged < 45 years, participants with a BMI ≤ 24, and participants with hypertension in Model 3. Details are shown in Suppl. Table 3.

## Discussion

A high ratio of LDL-C/HDL-C could significantly increase elevated CIMT risk regardless of discordance/concordance with TC, TGs, HDL-C, and LDL-C. Even a discordantly low ratio of LDL-C/HDL-C with normal HDL-C level and high level of LDL-C could significantly increase elevated CIMT risk. However, discordance of a low ratio of LDL-C/HDL-C with high TC, high TGs, abnormal HDL-C level and low LDL-C level was not significantly related with elevated CIMT risk.

Although the ratio of LDL-C/HDL-C is superior to single HDL-C and LDL-C in predicting atherosclerosis progression [10, 11], contradictory results have been reported [12–15]. Discordance of the ratio of LDL-C/HDL-C with various lipid parameters may explain the conflicting results to some extent. As far as we know, the study firstly focuses on the associations between the discordance of the ratio of LDL-C/HDL-C with alternative lipid parameters and elevated CIMT risk. A high ratio of LDL-C/HDL-C and discordantly low TC/TGs significantly increased elevated CIMT risk, whereas there was no significant association between a low ratio of LDL-C/HDL-C with discordantly high TC/TGs and elevated CIMT risk. The findings may imply the LDL-C/HDL-C ratio as a “risk enhancer” for the development of elevated CIMT. Sensitivity analyses and subgroup analyses further confirmed the robustness of the above results. However, the association of the discordance of a high ratio of LDL-C/HDL-C with low TC and elevated CIMT risk became nonsignificant in women and those with a BMI ≤ 24. The sex difference may be because that estrogen can inhibit the renin-angiotensin system (RAS) by simulating endothelium-derived relaxing factor release [21, 22], and thus protecting the vessel wall endothelial function. Obesity could increase blood pressure and subsequently increase peripheral vascular resistance [23], which may explain the nonsignificant association in the subgroups of individuals with a BMI ≤ 24. Moreover, the small sample size of discordance of a high ratio of LDL-C/HDL-C with low TC in women (*n* = 384) and individuals with a BMI ≤ 24 (*n* = 318) may also explain the nonsignificant associations in the subgroup analyses. The limited number of T2DM patients (*n* = 650) and even more limited number of T2DM patients with discordance of a high ratio of LDL-C/HDL-C with low TGs (*n* = 83) may explain the nonsignificant association between discordance of a high ratio of LDL-C/HDL-C with low TGs and elevated CIMT risk in the T2DM subgroup.

The significant association between discordantly low ratio of LDL-C/HDL-C with high LDL-C level and normal HDL-C level and elevated CIMT risk may explain the contradictory results of the relationship of the ratio of LDL-C/HDL-C and elevated CIMT risk [12–15]. In this study, discordance of a low ratio of LDL-C/HDL-C with high LDL-C level and normal HDL-C level existed in 9.9% of participants. Possibly, the action of antiatherogenic HDL particles may not be sufficient to prevent elevated CIMT in high LDL-C level population, although HDL should be more maturated in low LDL-C/HDL-C ratio population [24, 25]. Elevated CIMT risk was also increased in participants with a discordantly high ratio of LDL-C/HDL-C with low LDL-C level and abnormal HDL-C. The findings may be explained by the high level of smaller and cholesterol-depleted LDL particles at low HDL-C level, despite the low LDL-C level [26]. Low HDL-C is not enough to prevent elevated CIMT, but high level of smaller and cholesterol-depleted LDL particles could increase the risk of CIMT [27] because smaller particles are more easy enter the vascular intima, leading to subsequent subendothelial retention of LDL. Among participants with a low LDL-C/HDL-C ratio, none had discordantly high LDL-C and abnormal HDL-C; perhaps participants with high LDL-C level and abnormal HDL-C level would consistently have a high ratio of LDL-C/HDL-C.

Discordance of a high ratio of LDL-C/HDL-C with high LDL-C level and normal HDL-C level existed in 13.2% (1793/13,612) of participants, and discordance of a high ratio of LDL-C/HDL-C with low LDL-C level and normal HDL-C level existed in 3.5% of participants. A discordantly high ratio of LDL-C/HDL-C with normal HDL-C level and low/high LDL-C level could significantly increase elevated CIMT risk compared with the effect of a concordantly low LDL-C/HDL-C ratio with low LDL-C level and normal HDL-C level; the HR was higher in participants with a discordantly high ratio of LDL-C/HDL-C with normal HDL-C level and high LDL-C level. Although these individuals with a high LDL-C/HDL-C ratio had normal HDL-C levels, HDL may not consistently prevent atherosclerosis through the concentrations of HDL-C [28]. HDL could lose antioxidant effects or even gain a pro-inflammatory feature and become dysfunctional, thereby inducing atherosclerosis progression [29]. Moreover, there may be additional smaller-sized but less matured HDL particles because the ratio of LDL-C/HDL-C was closely related to HDL particles distribution. Increase in the ratio of LDL-C/HDL-C could block HDL maturation process and increase smaller-sized HDL particles numbers, thereby hampering cholesterol reverse transport from the peripheral tissues to steroidogenic organs and liver and inducing atherosclerosis progression [24, 25].

The sensitivity analyses further confirmed the above findings. According to the discordance status of the LDL-C/HDL-C ratio with LDL-C and HDL-C, participants were grouped into seven subgroups. The small sample sizes in the seven groups except for that in the reference group may explain the nonsignificant associations between a discordantly high ratio of LDL/HDL-C with low LDL-C and normal HDL-C and elevated CIMT risk in the subgroups of women, individuals aged < 45 years, individuals with a BMI ≤ 24, and individuals with hypertension in model 3. The nonsignificant associations between a discordantly low ratio of LDL-C/HDL-C with high LDL-C and normal HDL-C and elevated CIMT risk in the subgroups of women, individuals aged > 45 years, individuals with a BMI ≤ 24, and individuals with or without hypertension in Model 3 may be explained by the following: age related endothelial function impairment is an important factor in elevated CIMT, including the decrease in endothelium-derived relaxing factor and the increase in endothelium-derived vasoconstrictor factors with increasing age [30]; high blood pressure could also lead to vasomotor activity loss and permanent endothelial damage, and thereby increasing lipid permeability and CIMT risk [31]; obesity and sex effect on elevated CIMT risk were mentioned above.

### Study strengths and limitations

This cohort study with a large sample size could provide strong evidence for the significant associations between discordance of the LDL-C/HDL-C with TC, TGs, LDL-C, and HDL-C and the risk of elevated CIMT. However, limitations should also be considered when deriving conclusions. First, although this study was adjusted for numerous confounding factors, there may be potential residual confounding factors, as in the case with all observational studies. Second, because this was a dynamic cohort study, not all the participants finished the 7-year follow-up. However, a sensitivity analysis was performed by excluding patients with incident elevated CIMT diagnosed during the first two years of follow-up, which further validated the robustness of the results. Third, the discordance of the ratio of LDL-C/HDL-C with LDL-P was not estimated because of a large amount of missing data on LDL-P. Finally, most participants in this study were employees of local governmental organizations in China; thus, the results might represent the population with highly educated and employment.

## Conclusion

A high LDL-C/HDL-C ratio could increase elevated CIMT risk regardless of discordance/concordance with TC, TGs, LDL-C and HDL-C. A discordantly low LDL-C/HDL-C ratio with high LDL-C and normal HDL-C could also significantly increase elevated CIMT risk. The findings suggest the importance of both the LDL-C/HDL-C ratio and LDL-C in preventing elevated CIMT. More research is warranted to assess the effect of lowering the LDL-C/HDL-C ratio together with lowering LDL-C level treatment on the development of elevated CIMT.

## Supplementary information

**Additional file 1: Supplementary Table 1.** Association between LDL-C/HDL-C ratio concordance with TC and CIMT risk in the subgroup analyses. **Supplementary Table 2** Association between LDL-C/HDL-C ratio concordance with TG and CIMT risk in the subgroup analyses. **Supplementary Table 3** Association between LDL-C/HDL-C ratio concordance with LDL-C, HDL-C and CIMT risk in the subgroup analyses.

## Data Availability

All data generated or analyzed during this study are included in this published article and its supplementary information files.

## Abbreviations

CIMT: Carotid Intima-Media Thickness
CVD: Cardiovascular disease
LDL-C: Low-density lipoprotein cholesterol
HDL-C: High-density lipoprotein cholesterol
LDL-C/HDL-C: LDL-C to HDL-C
TC: Total cholesterol
TG: Triglycerides
WC: Waist circumference
BMI: Body Mass Index
WBC: White blood counts
