# Supplementary material for: LDL-cholesterol to HDL-cholesterol ratio discordance with lipid parameters and carotid intima-media thickness: a cohort study in China
Source: Lipids Health Dis. 2020 Jun 18;19:141. doi: 10.1186/s12944-020-01324-5 (PMC7302368; doi:10.1186/s12944-020-01324-5)
Supplement: Supplementary file 1 — Additional file 1: Supplementary Table 1. Association between LDL-C/HDL-C ratio concordance with TC and CIMT risk in the subgroup analyses. Supplementary Table 2 Association between LDL-C/HDL-C ratio concordance with TG and CIMT risk in the subgroup analyses. Supplementary Table 3 Association between LDL-C/HDL-C ratio concordance with LDL-C, HDL-C and CIMT risk in the subgroup analyses. [file 12944_2020_1324_MOESM1_ESM.docx]

**Suppl. Table 1 Association between LDL-C/HDL-C ratio concordance with TC and CIMT risk in the subgroup analyses**

|  | No. of participants/cases | Model 1^a^  (HR (95%CI)) | Model 2 ^b^  (HR (95%%CI)) | Model 3^c^  (HR (95%%CI)) |
| --- | --- | --- | --- | --- |
| **Men** |  |  |  |  |
| Concordantly low LDL-C/HDL-C ratio and low TC | 3760/526 | 1 | 1 | 1 |
| Discordantly low LDL-C/HDL-C ratio and high TC | 911/201 | 1.09 (0.92, 1.28) | 1.10 (0.93, 1.30) | 1.10 (0.93, 1.30) |
| Discordantly high LDL-C/HDL-C ratio and low TC | 1236/239 | 1.73 (1.48, 2.02) | 1.69 (1.44, 1.98) | 1.68 (1.43, 1.97) |
| Concordantly high LDL-C/HDL-C ratio and high TC | 1293/301 | 1.58 (1.37, 1.83) | 1.57 (1.35, 1.82) | 1.57 (1.35, 1.83) |
| **Women** |  |  |  |  |
| Concordantly low LDL-C/HDL-C ratio and low TC | 4359/397 | 1 | 1 | 1 |
| Discordantly low LDL-C/HDL-C ratio and high TC | 1149/182 | 0.88 (0.73, 1.06) | 0.93 (0.77, 1.12) | 0.94 (0.78, 1.13) |
| Discordantly high LDL-C/HDL-C ratio and low TC | 384/30 | 1.04 (0.72, 1.51) | 1.02 (0.70, 1.50) | 1.02 (0.69, 1.49) |
| Concordantly high LDL-C/HDL-C ratio and high TC | 519/119 | 1.88 (1.53, 2.32) | 1.91 (1.52, 2.39) | 1.88 (1.49, 2.36) |
| **Age ≤ 45 years** |  |  |  |  |
| Concordantly low LDL-C/HDL-C ratio and low TC | 5328/339 | 1 | 1 | 1 |
| Discordantly low LDL-C/HDL-C ratio and high TC | 916/95 | 1.22 (0.97, 1.53) | 1.22 (0.97, 1.53) | 1.24 (0.99, 1.56) |
| Discordantly high LDL-C/HDL-C ratio and low TC | 1004/105 | 1.85 (1.47, 2.31) | 1.68 (1.33, 2.12) | 1.71 (1.35, 2.16) |
| Concordantly high LDL-C/HDL-C ratio and high TC | 974/143 | 2.01 (1.64, 2.46) | 1.82 (1.47, 2.26) | 1.82 (1.47, 2.27) |
| **Age > 45 years** |  |  |  |  |
| Concordantly low LDL-C/HDL-C ratio and low TC | 2791/584 | 1 | 1 | 1 |
| Discordantly low LDL-C/HDL-C ratio and high TC | 1144/288 | 0.89 (0.77, 1.03) | 0.92 (0.80, 1.07) | 0.92 (0.79, 1.07) |
| Discordantly high LDL-C/HDL-C ratio and low TC | 616/164 | 1.43 (1.20, 1.71) | 1.48 (1.23, 1.77) | 1.44 (1.20, 1.73) |
| Concordantly high LDL-C/HDL-C ratio and high TC | 838/277 | 1.47 (1.27, 1.69) | 1.52 (1.30, 1.77) | 1.52 (1.30, 1.77) |
| **BMI ≤ 24** |  |  |  |  |
| Concordantly low LDL-C/HDL-C ratio and low TC | 4002/359 | 1 | 1 | 1 |
| Discordantly low LDL-C/HDL-C ratio and high TC | 908/143 | 0.85 (0.70, 1.05) | 0.88 (0.71, 1.08) | 0.89 (0.72, 1.09) |
| Discordantly high LDL-C/HDL-C ratio and low TC | 318/46 | 1.31 (0.96, 1.79) | 1.37 (0.99, 1.90) | 1.35 (0.97, 1.88) |
| Concordantly high LDL-C/HDL-C ratio and high TC | 335/67 | 1.46 (1.12, 1.91) | 1.61 (1.21, 2.13) | 1.62 (1.23, 2.15) |
| **BMI > 24** |  |  |  |  |
| Concordantly low LDL-C/HDL-C ratio and low TC | 3978/558 | 1 | 1 | 1 |
| Discordantly low LDL-C/HDL-C ratio and high TC | 1126/236 | 1.06 (0.91, 1.23) | 1.17 (1.00, 1.37) | 1.17 (1.01, 1.37) |
| Discordantly high LDL-C/HDL-C ratio and low TC | 1270/218 | 1.60 (1.36, 1.87) | 1.61 (1.37, 1.89) | 1.61 (1.37, 1.90) |
| Concordantly high LDL-C/HDL-C ratio and high TC | 1442/346 | 1.64 (1.43, 1.88) | 1.70 (1.48, 1.96) | 1.70 (1.47, 1.96) |
| **Hypertension** |  |  |  |  |
| Concordantly low LDL-C/HDL-C ratio and low TC | 1192/199 | 1 | 1 | 1 |
| Discordantly low LDL-C/HDL-C ratio and high TC | 412/101 | 1.17 (0.92, 1.49) | 1.23 (0.96, 1.58) | 1.2 (0.93, 1.54) |
| Discordantly high LDL-C/HDL-C ratio and low TC | 387/91 | 1.64 (1.28, 2.11) | 1.71 (1.33, 2.21) | 1.69 (1.31, 2.19) |
| Concordantly high LDL-C/HDL-C ratio and high TC | 493/132 | 1.62 (1.3, 2.02) | 1.64 (1.3, 2.06) | 1.62 (1.29, 2.04) |
| **Without hypertension** |  |  |  |  |
| Concordantly low LDL-C/HDL-C ratio and low TC | 6927/724 | 1 | 1 | 1 |
| Discordantly low LDL-C/HDL-C ratio and high TC | 1648/282 | 0.93 (0.81, 1.07) | 0.95 (0.82, 1.1) | 0.97 (0.84, 1.11) |
| Discordantly high LDL-C/HDL-C ratio and low TC | 1233/178 | 1.54 (1.3, 1.82) | 1.52 (1.28, 1.8) | 1.51 (1.27, 1.80) |
| Concordantly high LDL-C/HDL-C ratio and high TC | 1319/288 | 1.62 (1.41, 1.86) | 1.62 (1.4, 1.89) | 1.63 (1.4, 1.89) |
| **T2DM** |  |  |  |  |
| Concordantly low LDL-C/HDL-C ratio and low TC | 290/65 | 1 | 1 | 1 |
| Discordantly low LDL-C/HDL-C ratio and high TC | 104/21 | 0.97 (0.58, 1.6) | 1.14 (0.67, 1.91) | 0.96 (0.56, 1.64) |
| Discordantly high LDL-C/HDL-C ratio and low TC | 100/28 | 1.76 (1.12, 2.77) | 1.97 (1.21, 3.21) | 1.77 (1.09, 2.89) |
| Concordantly high LDL-C/HDL-C ratio and high TC | 154/40 | 1.44 (0.97, 2.15) | 1.78 (1.16, 2.75) | 1.92 (1.24, 2.98) |
| **Without T2DM** |  |  |  |  |
| Concordantly low LDL-C/HDL-C ratio and low TC | 7829/858 | 1 | 1 | 1 |
| Discordantly low LDL-C/HDL-C ratio and high TC | 1956/362 | 1 (0.88, 1.13) | 1.02 (0.9, 1.16) | 1.03 (0.91, 1.17) |
| Discordantly high LDL-C/HDL-C ratio and low TC | 1520/241 | 1.56 (1.35, 1.81) | 1.55 (1.33, 1.8) | 1.54 (1.33, 1.79) |
| Concordantly high LDL-C/HDL-C ratio and high TC | 1658/380 | 1.66 (1.47, 1.88) | 1.64 (1.44, 1.87) | 1.64 (1.44, 1.87) |

^a^ Model 1: adjusted for age, gender at baseline.

^b^ Model 2: adjusted for age, gender, BMI, smoking, drinking, WC, TC, TG, creatinine, lipid-lowering medications, T2DM, hypertension, and fatty liver at baseline.

^c^ Model 3: adjusted for age, gender, BMI, smoking, drinking, WC, TC, TG, creatinine, WBC, lipid-lowering medications, T2DM, hypertension, and fatty liver at baseline.

**Suppl. Table 2 Association between LDL-C/HDL-C ratio concordance with TG and CIMT risk in the subgroup analyses**

|  | No. of participants/cases | Model 1 ^a^  (HR (95%CI)) | Model 2 ^b^  (HR (95%%CI)) | Model 3 ^c^  (HR (95%%CI)) |
| --- | --- | --- | --- | --- |
| **Men** |  |  |  |  |
| Concordantly low LDL-C/HDL-C ratio and low TG | 3223/491 | 1 | 1 | 1 |
| Discordantly low LDL-C/HDL-C ratio and high TG | 1448/236 | 1.02 (0.87, 1.19) | 0.95 (0.81, 1.12) | 0.93 (0.79, 1.10) |
| Discordantly high LDL-C/HDL-C ratio and low TG | 1098/226 | 1.69 (1.44, 1.98) | 1.61 (1.37, 1.89) | 1.58 (1.35, 1.86) |
| Concordantly high LDL-C/HDL-C ratio and TG | 1431/314 | 1.57 (1.36, 1.81) | 1.46 (1.25, 1.70) | 1.45 (1.24, 1.69) |
| **Women** |  |  |  |  |
| Concordantly low LDL-C/HDL-C ratio and low TG | 4985/504 | 1 | 1 | 1 |
| Discordantly low LDL-C/HDL-C ratio and high TG | 523/75 | 0.84 (0.66, 1.08) | 0.69 (0.52, 0.9) | 0.68 (0.52, 0.89) |
| Discordantly high LDL-C/HDL-C ratio and low TG | 550/83 | 1.68 (1.33, 2.12) | 1.54 (1.21, 1.97) | 1.53 (1.19, 1.96) |
| Concordantly high LDL-C/HDL-C ratio and TG | 353/66 | 1.61 (1.25, 2.09) | 1.28 (0.97, 1.7) | 1.24 (0.93, 1.64) |
| **Age ≤ 45 years** |  |  |  |  |
| Concordantly low LDL-C/HDL-C ratio and low TG | 5216/341 | 1 | 1 | 1 |
| Discordantly low LDL-C/HDL-C ratio and high TG | 1028/93 | 1.01 (0.8, 1.28) | 0.83 (0.65, 1.07) | 0.84 (0.65, 1.08) |
| Discordantly high LDL-C/HDL-C ratio and low TG | 943/111 | 1.77 (1.42, 2.21) | 1.50 (1.19, 1.90) | 1.52 (1.21, 1.92) |
| Concordantly high LDL-C/HDL-C ratio and TG | 1035/137 | 1.95 (1.57, 2.41) | 1.55 (1.23, 1.97) | 1.56 (1.23, 1.98) |
| **Age > 45 years** |  |  |  |  |
| Concordantly low LDL-C/HDL-C ratio and low TG | 2992/654 | 1 | 1 | 1 |
| Discordantly low LDL-C/HDL-C ratio and high TG | 943/218 | 0.94 (0.81, 1.10) | 0.87 (0.74, 1.03) | 0.85 (0.72, 1.00) |
| Discordantly high LDL-C/HDL-C ratio and low TG | 705/198 | 1.62 (1.38, 1.91) | 1.58 (1.34, 1.87) | 1.54 (1.30, 1.82) |
| Concordantly high LDL-C/HDL-C ratio and TG | 749/243 | 1.39 (1.19, 1.61) | 1.31 (1.11, 1.54) | 1.29 (1.10, 1.52) |
| **BMI ≤ 24** |  |  |  |  |
| Concordantly low LDL-C/HDL-C ratio and low TG | 4540/459 | 1 | 1 | 1 |
| Discordantly low LDL-C/HDL-C ratio and high TG | 370/43 | 0.79 (0.57, 1.09) | 0.76 (0.55, 1.07) | 0.77 (0.55, 1.07) |
| Discordantly high LDL-C/HDL-C ratio and low TG | 435/72 | 1.49 (1.15, 1.92) | 1.50 (1.15, 1.94) | 1.50 (1.15, 1.94) |
| Concordantly high LDL-C/HDL-C ratio and TG | 218/41 | 1.33 (0.96, 1.84) | 1.36 (0.97, 1.91) | 1.35 (0.96, 1.91) |
| **BMI > 24** |  |  |  |  |
| Concordantly low LDL-C/HDL-C ratio and low TG | 3539/530 | 1 | 1 | 1 |
| Discordantly low LDL-C/HDL-C ratio and high TG | 1565/264 | 0.95 (0.82, 1.11) | 0.88 (0.76, 1.03) | 0.86 (0.74, 1.01) |
| Discordantly high LDL-C/HDL-C ratio and low TG | 1178/230 | 1.65 (1.41, 1.93) | 1.60 (1.36, 1.87) | 1.57 (1.34, 1.84) |
| Concordantly high LDL-C/HDL-C ratio and TG | 1534/334 | 1.52 (1.32, 1.75) | 1.41 (1.22, 1.64) | 1.39 (1.20, 1.61) |
| **Hypertension** |  |  |  |  |
| Concordantly low LDL-C/HDL-C ratio and low TG | 973/180 | 1 | 1 | 1 |
| Discordantly low LDL-C/HDL-C ratio and high TG | 631/120 | 0.89 (0.71, 1.13) | 0.81 (0.63, 1.03) | 0.77 (0.6, 0.99) |
| Discordantly high LDL-C/HDL-C ratio and low TG | 341/78 | 1.5 (1.14, 1.96) | 1.49 (1.14, 1.96) | 1.41 (1.07, 1.86) |
| Concordantly high LDL-C/HDL-C ratio and TG | 539/145 | 1.47 (1.18, 1.84) | 1.32 (1.04, 1.67) | 1.31 (1.03, 1.66) |
| **Without hypertension** |  |  |  |  |
| Concordantly low LDL-C/HDL-C ratio and low TG | 7235/815 | 1 | 1 | 1 |
| Discordantly low LDL-C/HDL-C ratio and high TG | 1340/191 | 0.95 (0.81, 1.11) | 0.86 (0.73, 1.02) | 0.86 (0.73, 1.02) |
| Discordantly high LDL-C/HDL-C ratio and low TG | 1307/231 | 1.67 (1.44, 1.94) | 1.6 (1.37, 1.86) | 1.60 (1.37, 1.87) |
| Concordantly high LDL-C/HDL-C ratio and TG | 1245/235 | 1.53 (1.31, 1.78) | 1.39 (1.19, 1.64) | 1.37 (1.16, 1.62) |
| **T2DM** |  |  |  |  |
| Concordantly low LDL-C/HDL-C ratio and low TG | 206/53 | 1 | 1 | 1 |
| Discordantly low LDL-C/HDL-C ratio and high TG | 188/33 | 0.82 (0.53, 1.26) | 0.57 (0.34, 0.95) | 0.49 (0.29, 0.82) |
| Discordantly high LDL-C/HDL-C ratio and low TG | 83/21 | 1.53 (0.92, 2.57) | 1.4 (0.81, 2.42) | 1.34 (0.77, 2.32) |
| Concordantly high LDL-C/HDL-C ratio and TG | 171/47 | 1.4 (0.94, 2.09) | 1.25 (0.8, 1.97) | 1.23 (0.78, 1.94) |
| **Without T2DM** |  |  |  |  |
| Concordantly low LDL-C/HDL-C ratio and low TG | 8002/942 | 1 | 1 | 1 |
| Discordantly low LDL-C/HDL-C ratio and high TG | 1783/278 | 0.98 (0.85, 1.12) | 0.88 (0.76, 1.02) | 0.88 (0.76, 1.01) |
| Discordantly high LDL-C/HDL-C ratio and low TG | 1565/288 | 1.68 (1.47, 1.93) | 1.62 (1.41, 1.86) | 1.6 (1.39, 1.84) |
| Concordantly high LDL-C/HDL-C ratio and TG | 1613/333 | 1.55 (1.36, 1.77) | 1.38 (1.2, 1.59) | 1.37 (1.19, 1.58) |

^a^ Model 1: adjusted for age, gender at baseline.

^b^ Model 2: adjusted for age, gender, BMI, smoking, drinking, WC, TC, TG, creatinine, lipid-lowering medications, T2DM, hypertension, and fatty liver at baseline.

^c^ Model 3: adjusted for age, gender, BMI, smoking, drinking, WC, TC, TG, creatinine, WBC, lipid-lowering medications, T2DM, hypertension, and fatty liver at baseline.

**Suppl. Table 3 Association between LDL-C/HDL-C ratio concordance with LDL-C, HDL-C and CIMT risk in the subgroup analyses**

|  | No. of participants/cases | Model 1 ^a^  (HR (95%CI)) | Model 2 ^b^  (HR (95%%CI)) | Model 3 ^c^  (HR (95%%CI)) |
| --- | --- | --- | --- | --- |
| **Men** |  |  |  |  |
| Concordantly low LDL-C/HDL-C ratio, low LDL-C and normal HDL-C | 3566/534 | 1 | 1 | 1 |
| Discordantly low LDL-C/HDL-C ratio, high LDL-C and normal HDL-C | 569/127 | 1.23 (1.01, 1.49) | 1.28 (1.05, 1.56) | 1.26 (1.04, 1.55) |
| Discordantly low LDL/HDL-C ratio, low LDL-C and abnormal HDL-C | 537/67 | 1.04 (0.81, 1.34) | 1.01 (0.78, 1.30) | 0.97 (0.75, 1.26) |
| Discordantly high LDL/HDL-C ratio, low LDL-C and normal HDL-C | 354/61 | 1.43 (1.10, 1.87) | 1.41 (1.08, 1.84) | 1.40 (1.07, 1.83) |
| Discordantly high LDL/HDL-C ratio, low LDL-C and abnormal HDL-C | 619/127 | 1.61 (1.33, 1.96) | 1.57 (1.28, 1.93) | 1.57 (1.28, 1.92) |
| Discordantly high LDL/HDL-C ratio, high LDL-C and normal HDL-C | 1196/283 | 1.67 (1.44, 1.93) | 1.66 (1.43, 1.93) | 1.66 (1.43, 1.93) |
| Concordantly high LDL/HDL-C ratio, high LDL-C and abnormal HDL-C | 360/69 | 2.1 (1.63, 2.71) | 1.96 (1.51, 2.55) | 1.86 (1.42, 2.42) |
| **Women** |  |  |  |  |
| Concordantly low LDL-C/HDL-C ratio, low LDL-C and normal HDL-C | 4592/443 | 1 | 1 | 1 |
| Discordantly low LDL-C/HDL-C ratio, high LDL-C and normal HDL-C | 774/119 | 1.15 (0.94, 1.41) | 1.19 (0.97, 1.47) | 1.19 (0.96, 1.47) |
| Discordantly low LDL/HDL-C ratio, low LDL-C and abnormal HDL-C | 142/17 | 1.19 (0.73, 1.93) | 1.11 (0.67, 1.85) | 1.17 (0.71, 1.95) |
| Discordantly high LDL/HDL-C ratio, low LDL-C and normal HDL-C | 118/16 | 1.54 (0.93, 2.53) | 1.59 (0.96, 2.63) | 1.59 (0.96, 2.63) |
| Discordantly high LDL/HDL-C ratio, low LDL-C and abnormal HDL-C | 130/10 | 1.01 (0.54, 1.89) | 1.00 (0.53, 1.89) | 0.98 (0.52, 1.86) |
| Discordantly high LDL/HDL-C ratio, high LDL-C and normal HDL-C | 397/118 | 1.91 (1.56, 2.35) | 1.88 (1.5, 2.34) | 1.85 (1.48, 2.31) |
| Concordantly high LDL/HDL-C ratio, high LDL-C and abnormal HDL-C | 58/5 | 1.63 (0.68, 3.95) | 1.05 (0.34, 3.3) | 1.04 (0.33, 3.24) |
| **Age ≤ 45 years** |  |  |  |  |
| Concordantly low LDL-C/HDL-C ratio, low LDL-C and normal HDL-C | 5218/326 | 1 | 1 | 1 |
| Discordantly low LDL-C/HDL-C ratio, high LDL-C and normal HDL-C | 609/69 | 1.63 (1.25, 2.11) | 1.72 (1.32, 2.23) | 1.74 (1.34, 2.26) |
| Discordantly low LDL/HDL-C ratio, low LDL-C and abnormal HDL-C | 418/40 | 1.42 (1.02, 1.99) | 1.3 (0.93, 1.82) | 1.28 (0.90, 1.80) |
| Discordantly high LDL/HDL-C ratio, low LDL-C and normal HDL-C | 288/26 | 1.48 (0.99, 2.22) | 1.36 (0.91, 2.04) | 1.38 (0.92, 2.07) |
| Discordantly high LDL/HDL-C ratio, low LDL-C and abnormal HDL-C | 454/50 | 1.95 (1.44, 2.64) | 1.7 (1.24, 2.34) | 1.72 (1.25, 2.36) |
| Discordantly high LDL/HDL-C ratio, high LDL-C and normal HDL-C | 965/138 | 2.17 (1.77, 2.66) | 1.99 (1.61, 2.47) | 2.01 (1.62, 2.50) |
| Concordantly high LDL/HDL-C ratio, high LDL-C and abnormal HDL-C | 271/34 | 2.47 (1.72, 3.55) | 2.07 (1.41, 3.03) | 2.01 (1.36, 2.96) |
| **Age > 45 years** |  |  |  |  |
| Concordantly low LDL-C/HDL-C ratio, low LDL-C and normal HDL-C | 2940/651 | 1 | 1 | 1 |
| Discordantly low LDL-C/HDL-C ratio, high LDL-C and normal HDL-C | 734/177 | 1.05 (0.89, 1.24) | 1.05 (0.88, 1.25) | 1.04 (0.87, 1.23) |
| Discordantly low LDL/HDL-C ratio, low LDL-C and abnormal HDL-C | 261/44 | 0.88 (0.65, 1.19) | 0.86 (0.63, 1.18) | 0.85 (0.62, 1.17) |
| Discordantly high LDL/HDL-C ratio, low LDL-C and normal HDL-C | 184/51 | 1.43 (1.07, 1.9) | 1.47 (1.1, 1.97) | 1.46 (1.09, 1.95) |
| Discordantly high LDL/HDL-C ratio, low LDL-C and abnormal HDL-C | 295/87 | 1.36 (1.08, 1.71) | 1.40 (1.10, 1.77) | 1.38 (1.08, 1.75) |
| Discordantly high LDL/HDL-C ratio, high LDL-C and normal HDL-C | 828/263 | 1.54 (1.33, 1.78) | 1.56 (1.34, 1.82) | 1.56 (1.34, 1.81) |
| Concordantly high LDL/HDL-C ratio, high LDL-C and abnormal HDL-C | 147/40 | 1.86 (1.35, 2.57) | 1.77 (1.27, 2.47) | 1.67 (1.19, 2.34) |
| **BMI ≤ 24** |  |  |  |  |
| Concordantly low LDL-C/HDL-C ratio, low LDL-C and normal HDL-C | 4199/394 | 1 | 1 | 1 |
| Discordantly low LDL-C/HDL-C ratio, high LDL-C and normal HDL-C | 590/102 | 1.22 (0.98, 1.53) | 1.23 (0.98, 1.54) | 1.22 (0.97, 1.54) |
| Discordantly low LDL/HDL-C ratio, low LDL-C and abnormal HDL-C | 122/7 | 0.61 (0.29, 1.30) | 0.57 (0.27, 1.22) | 0.58 (0.27, 1.24) |
| Discordantly high LDL/HDL-C ratio, low LDL-C and normal HDL-C | 105/13 | 1.11 (0.64, 1.93) | 1.12 (0.64, 1.97) | 1.14 (0.65, 2.00) |
| Discordantly high LDL/HDL-C ratio, low LDL-C and abnormal HDL-C | 103/17 | 1.43 (0.87, 2.36) | 1.51 (0.90, 2.52) | 1.48 (0.88, 2.48) |
| Discordantly high LDL/HDL-C ratio, high LDL-C and normal HDL-C | 382/76 | 1.62 (1.26, 2.08) | 1.76 (1.35, 2.29) | 1.75 (1.35, 2.28) |
| Concordantly high LDL/HDL-C ratio, high LDL-C and abnormal HDL-C | 63/7 | 1.45 (0.68, 3.07) | 1.38 (0.64, 2.95) | 1.41 (0.66, 3.01) |
| **BMI > 24** |  |  |  |  |
| Concordantly low LDL-C/HDL-C ratio, low LDL-C and normal HDL-C | 3828/576 | 1 | 1 | 1 |
| Discordantly low LDL-C/HDL-C ratio, high LDL-C and normal HDL-C | 730/142 | 1.17 (0.97, 1.4) | 1.22 (1.01, 1.47) | 1.21 (1.00, 1.46) |
| Discordantly low LDL/HDL-C ratio, low LDL-C and abnormal HDL-C | 546/76 | 1.10 (0.87, 1.40) | 1.11 (0.87, 1.42) | 1.08 (0.85, 1.39) |
| Discordantly high LDL/HDL-C ratio, low LDL-C and normal HDL-C | 358/64 | 1.51 (1.17, 1.96) | 1.51 (1.16, 1.96) | 1.50 (1.15, 1.94) |
| Discordantly high LDL/HDL-C ratio, low LDL-C and abnormal HDL-C | 635/116 | 1.46 (1.19, 1.78) | 1.51 (1.23, 1.86) | 1.50 (1.22, 1.85) |
| Discordantly high LDL/HDL-C ratio, high LDL-C and normal HDL-C | 1378/321 | 1.71 (1.49, 1.96) | 1.72 (1.49, 1.98) | 1.71 (1.48, 1.97) |
| Concordantly high LDL/HDL-C ratio, high LDL-C and abnormal HDL-C | 341/63 | 2.02 (1.56, 2.63) | 1.98 (1.52, 2.59) | 1.86 (1.42, 2.44) |
| **Hypertension** |  |  |  |  |
| Concordantly low LDL-C/HDL-C ratio, low LDL-C and normal HDL-C | 1175/217 | 1 | 1 | 1 |
| Discordantly low LDL-C/HDL-C ratio, high LDL-C and normal HDL-C | 231/54 | 1.38 (1.03, 1.86) | 1.35 (0.99, 1.84) | 1.31 (0.96, 1.79) |
| Discordantly low LDL/HDL-C ratio, low LDL-C and abnormal HDL-C | 198/29 | 0.96 (0.65, 1.41) | 0.88 (0.59, 1.32) | 0.88 (0.59, 1.32) |
| Discordantly high LDL/HDL-C ratio, low LDL-C and normal HDL-C | 128/27 | 1.40(0.94, 2.10) | 1.45 (0.97, 2.18) | 1.43 (0.96, 2.15) |
| Discordantly high LDL/HDL-C ratio, low LDL-C and abnormal HDL-C | 199/50 | 1.53 (1.12, 2.09) | 1.54 (1.11, 2.13) | 1.55 (1.12, 2.14) |
| Discordantly high LDL/HDL-C ratio, high LDL-C and normal HDL-C | 451/125 | 1.65 (1.32, 2.06) | 1.62 (1.29, 2.03) | 1.60 (1.27, 2.01) |
| Concordantly high LDL/HDL-C ratio, high LDL-C and abnormal HDL-C | 102/21 | 2.12 (1.35, 3.33) | 2.02 (1.27, 3.21) | 1.97 (1.24, 3.14) |
| **Without hypertension** |  |  |  |  |
| Concordantly low LDL-C/HDL-C ratio, low LDL-C and normal HDL-C | 6983/760 | 1 | 1 | 1 |
| Discordantly low LDL-C/HDL-C ratio, high LDL-C and normal HDL-C | 1112/192 | 1.15 (0.98, 1.35) | 1.15 (0.97, 1.35) | 1.15 (0.98, 1.36) |
| Discordantly low LDL/HDL-C ratio, low LDL-C and abnormal HDL-C | 481/55 | 1.11 (0.84, 1.46) | 1.09 (0.83, 1.45) | 1.06 (0.80, 1.42) |
| Discordantly high LDL/HDL-C ratio, low LDL-C and normal HDL-C | 344/50 | 1.47 (1.11, 1.97) | 1.50 (1.12, 2.00) | 1.50 (1.12, 2.01) |
| Discordantly high LDL/HDL-C ratio, low LDL-C and abnormal HDL-C | 550/87 | 1.49 (1.19, 1.87) | 1.46 (1.15, 1.84) | 1.44 (1.13, 1.82) |
| Discordantly high LDL/HDL-C ratio, high LDL-C and normal HDL-C | 1342/276 | 1.72 (1.49, 1.98) | 1.72 (1.48, 1.99) | 1.72 (1.48, 1.99) |
| Concordantly high LDL/HDL-C ratio, high LDL-C and abnormal HDL-C | 316/53 | 1.99 (1.50, 2.63) | 1.92 (1.43, 2.59) | 1.82 (1.35, 2.47) |
| **Sensitivity analysis by excluding T2DM at baseline ^#^** |  |  |  |  |
| Concordantly low LDL-C/HDL-C ratio, low LDL-C and normal HDL-C | 7882/910 | 1 | 1 | 1 |
| Discordantly low LDL-C/HDL-C ratio, high LDL-C and normal HDL-C | 1287/238 | 1.23 (1.07, 1.42) | 1.22 (1.05, 1.41) | 1.22 (1.06, 1.42) |
| Discordantly low LDL/HDL-C ratio, low LDL-C and abnormal HDL-C | 616/72 | 1.06 (0.83, 1.35) | 1.04 (0.81, 1.33) | 1.02 (0.79, 1.31) |
| Discordantly high LDL/HDL-C ratio, low LDL-C and normal HDL-C | 441/71 | 1.49 (1.17, 1.9) | 1.50 (1.17, 1.91) | 1.50 (1.17, 1.91) |
| Discordantly high LDL/HDL-C ratio, low LDL-C and abnormal HDL-C | 696/120 | 1.50 (1.23, 1.82) | 1.45 (1.19, 1.78) | 1.45 (1.18, 1.77) |
| Discordantly high LDL/HDL-C ratio, high LDL-C and normal HDL-C | 1662/369 | 1.77 (1.56, 2.00) | 1.74 (1.53, 1.97) | 1.73 (1.52, 1.96) |
| Concordantly high LDL/HDL-C ratio, high LDL-C and abnormal HDL-C | 379/61 | 1.93 (1.49, 2.51) | 1.85 (1.41, 2.43) | 1.80 (1.37, 2.37) |

^a^ Model 1: adjusted for age, gender at baseline.

^b^ Model 2: adjusted for age, gender, BMI, smoking, drinking, WC, TC, TG, creatinine, lipid-lowering medications, T2DM, hypertension, and fatty liver at baseline.

^c^ Model 3: adjusted for age, gender, BMI, smoking, drinking, WC, TC, TG, creatinine, WBC, lipid-lowering medications, T2DM, hypertension, and fatty liver at baseline.

**^#^** Subgroup analysis stratifying by T2DM was not conducted because of small sample size of T2DM at baseline.
